# Diagnostic and prognostic relevance of *RAF1* gene in acute myeloid leukemia

**DOI:** 10.1016/j.lrr.2025.100528

**Published:** 2025-07-10

**Authors:** Seyedeh Kosar Rahimpour, Yasin Mirazimi, Seyedeh Zahra Shahrokhi, Mohammad Sayyadi, Maryam Kargar, Mohammad Rafiee

**Affiliations:** a Department of Medical Laboratory Sciences, School of Paramedical Sciences, Zanjan University of Medical Sciences, Zanjan, Iran; b Department of Biochemistry, Faculty of Medicine, Aja University of Medical Sciences, Tehran, Iran; c Department of Medical Laboratory Sciences, School of Allied Medical Sciences, Arak University of Medical Sciences, Arak, Iran; d Department of Laboratory Hematology and Blood Bank, School of Allied Medical Sciences, Shahid Beheshti University of Medical Sciences, Tehran, Iran; e Department of Medical Laboratory Sciences, School of Paramedical Sciences, Hamadan University of Medical Sciences, Hamadan, Iran

**Keywords:** Acute myeloid leukemia, Diagnosis, Prognosis, RAF1, miR-146b-3p, Circ-RPL15

## Abstract

Acute Myeloid Leukemia (AML) demands precise diagnostic and prognostic tools for accurate detection and personalized-treatment strategies. Using Real-Time PCR, we investigated the RAF1, miR-146 b-3p, and Circ-RPL15 network expression in AML. Because RAF1 is a crucial gene involved in various ways, it is considered a potential biomarker for prognosis and diagnosis. While no significant correlation was found within the network, RAF1 expression showed a marked difference between groups. ROC curve analysis for RAF1 demonstrated promising diagnostic performance, and our bioinformatics investigations supported its strong prognostic potential. Identifying such biomarkers can aid in developing better management based on patients' characteristics.

## Highlights

• Increased *RAF1* gene expression in AML.
• Significant diagnostic potential of *RAF1* gene for AML.
• Strong prognostic potential of *RAF1* gene for AML.

## 1 Introduction

AML is a heterogeneous blood malignancy characterized by the rapid proliferation of immature myeloid cells, known as blasts, in the bone marrow [1]. These abnormal cells then infiltrate the bloodstream. A study published in 2024 reported a significant increase in AML cases, particularly among the 60–89 age group, with 61,559 new cases and 53,620 related deaths in 2019 alone. Over the past three decades, the incidence and mortality rates of AML have risen by 1.67 and 1.57 per 100,000 individuals, respectively [2]. The aggressive nature of this disease is evident, with a five-year survival rate of only 39.4 % reported in a study involving 222 AML patients [3]. AML diagnosis typically involves a combination of clinical evaluation, blood tests, and bone marrow biopsy to confirm the presence of myeloid blasts [4]. The escalating mortality rates associated with AML underscore the urgent need for further research to uncover the underlying causes and develop more effective treatment strategies. Numerous studies have highlighted the critical role of signaling pathways in AML, such as the PI3K-Akt-mTOR and NOTCH signaling pathways [5,6].

The RAF1 gene encodes a protein that is part of the RAS/MAPK signaling pathway, which plays a pivotal role in cell growth and proliferation. RAF1 is classified as an oncogene, meaning that mutations in this gene can transform normal cells into cancerous ones [7,8]. Huang and colleagues conducted a comprehensive study of RAF1 and its interactions with other proteins, revealing that RAF1 interacts with 198 proteins involved in various signaling pathways and biological processes, including those associated with cancer [9]. MicroRNAs (miRNAs) are small non-coding RNA molecules that play a crucial role in gene regulation. Studies have shown that RAF1 and miR-146b-3p interact, with miR-146b-3p regulating the expression of RAF1 [10]. Circ-RPL15 is a circular RNA that can act as a "sponge" for miR-146b-3p, sequestering it and preventing it from binding to target mRNAs like RAF1 [11]. In modern medicine, the identification of prognostic biomarkers is recognized as a cornerstone in the management and prognosis of diseases. These markers play a critical role in clinical decision-making by providing valuable insights into disease progression, treatment response, and the likelihood of recurrence or patient survival. Recent advances in omics technologies and bioinformatics have significantly enhanced the ability to discover and validate such biomarkers with greater accuracy and speed. In many chronic and malignant conditions, including various cancers, prognostic markers help stratify patients based on their predicted outcomes, thereby paving the way for more personalized therapeutic strategies. Thus, research on prognostic biomarkers holds not only academic but also substantial clinical value, representing a crucial step toward the realization of precision medicine

This study aims to investigate the expression levels, correlation and diagnostic value of the RAF1, miR-146b-3p, and Circ-RPL15 network in AML patients, compared to healthy controls. Subsequently, the analysis will focus on assessing the prognostic implications of RAF1, aiming to determine its value as a biomarker for clinical decision-making and better understand its potential impact on disease progression and patient prognosis.

## 2 Material and methods

### 2.1 Participants and sample collection

• **Recruitment:** 40 untreated AML patients and 32 healthy individuals were recruited as controls.
• **Informed Consent:** Participants provided written informed consent after receiving adequate information about the study.
• **Ethics Approval:** The study was approved by the ethics committee of Zanjan University of Medical Sciences, Iran.
• **Sample Collection:** Blood and bone marrow samples were collected in EDTA tubes.

### 2.2 RNA extraction and cDNA synthesis

• **RNA Extraction:** Total RNA was extracted from peripheral blood mononuclear cells (PBMCs) and bone marrow mononuclear cells (BMMCs) using the TRIzol reagent (Thermo Fisher Scientific, Waltham, MA, USA). RNA quality and quantity were assessed using a nanodrop spectrophotometer (Micro UV–VIS Spectrophotometer, BOECO N-1C, Germany) and gel electrophoresis.
• **cDNA Synthesis:** cDNA was synthesized from total RNA using a reverse transcriptase kit (ExcelRT SMOBio, South Korea) and a universal stem-loop primer for microRNA.

### 2.3 Real-Time quantitative PCR (qPCR)

• **qPCR Assay:** qPCR was performed using SYBR Green chemistry. The total volume per reaction was assumed to be 25-μl (12.5 μl Master mix, 0.5 μl of each primer, 3 μl cDNA and 8.5 μl DEPS water. The expression levels of RAF1, Circ-RPL15, and miR-146b-3p were quantified relative to reference genes (B2-M for RAF1 and Circ-RPL15, and Snord47 for miR-146b-3p). Finally, it was performed in Applied Biosystems Step One Plus (Thermo Fisher Scientific, CA, USA). The sequence of primers and the efficiency obtained with the help of linreg PCR software are shown in Table 1. The qPCR program was as follows: Holding phase: 15 min at a temperature of 95 degrees, cycling phase: 40 cycles of repetition of this program: 15 s at 95 degrees, 25 s at annealing temperature, 25 s as extension time at 72 degrees and 5 min of the final extension that only one cycle was done, Melt curve: 15 s at 99 degrees, 1 min at 60 degrees, 15 s at 95 degrees, only one cycle was done. In order to avoid bias during cDNA synthesis and Real-Time PCR, patient and control samples were blinded.

**Table 1:** Primers sequences.

Gene Name	Sequences of primers (5′ → 3′)	Optimum annealing ™	Mean PCR efficiency
RAF1	F: GGGAGCTTGGAAGACGATCAG R: ACACGGATAGTGTTGCTTGTC	60	1.90
miR-146b-3p	F: GAATAGCCCTGTGGACTCA R: CGAGGAAGAAGACGGAAGAAT	58	1.87
Circ-RPL15	F: ACGGCAAGCCTGTCCATCAT R: GCAGCGGACCCTCAGAAGAA	62	1.93
B2-M	F: AGGCTATCCAGCGTACTCCA R: TCATCCAATCCAAATGCGGC	60	1.93
Snord47	F: CCAATGATGTAATGATTCTGCCA R: CGAGGAAGAAGACGGAAGAAT	61	1.88
USLP	GAAAGAAGGCGAGGAGCAGATCGAGGAAGAAGACGGAAGAATGTGCGTCTCGCCTTCTTTCNNNNNN
• **Data Analysis:** Relative fold change (RFC) was calculated to compare gene expression between the AML and control groups using this formula.
  $$\text{RFC}={\left(E\;\text{Target}\right)}^{\Delta \text{CT}\;\text{Target}\;\left(\text{control}-\text{sample}\right)}/{\left(E\;\text{Reference}\right)}^{\Delta \text{CT}\;\text{Reference}\left(\text{control}-\text{sample}\right)}$$

### 2.4 Statistical analysis

• **Software:** SPSS Statistics 25.0(IBM, Armonk, NY), STATA version 15(StataCorp, College Station, TX), and R 4.4.2 (pROC package) were used for statistical analysis.
• **Tests:**
  ○ Two-independent sample *t*-test or Mann-Whitney U test for comparing continuous variables between groups.
  ○ Receiver operating characteristic (ROC) curve analysis to assess the diagnostic value of genes.
• **Significance:** A p-value <0.05 was considered statistically significant.
• **Survival Analysis:** In this study, we aimed to investigate the prognostic significance of RAF1 in AML. Given the unavailability of long-term patient follow-up data, we utilized publicly available genomic datasets and performed statistical modeling, and validation of results through cross-validation techniques. This approach allowed us to derive insights into the potential role of RAF1 as a prognostic marker, despite the limitations of clinical data. The Kaplan-Meier Plotter Database (https://kmplot.com/analysis/) was utilized to evaluate the prognostic significance of RAF1 in patients suffering from AML. The Kaplan-Meier Plotter serves as an online tool that provides relapse-free survival (RFS) and overall survival (OS) data derived from the Gene Expression Omnibus (GEO), the European Genome-phenome Archive (EGA), and The Cancer Genome Atlas (TCGA) databases, thereby facilitating the assessment of the prognostic implications of specific genes. The figure presented (Fig. 2) include the log-rank P value and the hazard ratio (HR) accompanied by 95 % confidence intervals. In instances where multiple probe sets targeted the same gene, the probe set exhibiting the highest standard deviation (SD) was chosen for analysis [12]. Patients were dichotomized into high and low expression groups based on the median expression value for each gene, as performed by the Kaplan-Meier Plotter tool. This method ensures consistency across large cohort comparisons

## 3 Results

In this study, 41.7 % of the control group participants was female, while 58.3 % was male. In the patient group, these statistics were 51.5 % females and 48.5 % males, respectively.

### 3.1 Comparison of baseline and clinicopathological information between control and patient groups

Table 2 presents the differences in clinicopathological characteristics between AML patients and healthy controls. Statistical analysis revealed significant differences between the AML patients and healthy controls in parameters such as RBC count, WBC count, PLT count, HB level, and HCT percentage. However, no significant differences were observed between the two groups concerning age and gender.

**Table 2:** Demographic and clinicopathological data of participants.

Variables		Control	Patient	P-value
Sex	Female	10 (41.7 %)	17 (51.5 %)	0.46
	Male	14 (58.3 %0)	16 (48.5 %)	
Age	Mean±SD	40.50±11.74	46.12±13.1	0.10
	Median (Q_1_, Q_3_)	41	46	
	Min, Max	20–67	23–67	
WBC counts	Mean±SD	6.72±1.77	62.18±68.52	<0.001
	Median (Q_1_, Q_3_)	6.35	38	
	Min, Max	4.4–11.2	2.2–220	
RBC counts	Mean±SD	5.34±0.65	2.61±0.88	<0.001
	Median (Q_1_, Q_3_)	5.43	2.56	
	Min, Max	4.09–6.32	1.18–4.37	
HB	Mean±SD	15.07±1.69	8.02±2.40	<0.001
	Median (Q_1_, Q_3_)	14.95	7.4	
	Min, Max	11.9–18.6	3.6–11.7	
HCT	Mean±SD	45.19±4.39	23.72±7.10	<0.001
	Median (Q_1_, Q_3_)	44.90	22.50	
	Min, Max	36.4–51.9	10–37	
Platelet counts	Mean±SD	213.91±42.36	68.63±77.48	<0.001
	Median (Q_1_, Q_3_)	205.5	46.00	
	Min, Max	143–313	4–357	
RFC (RAF1)	Mean±SD	1.38±3.19	376.90±1162.10	<0.001
	Median (Q_1_, Q_3_)	0.20	7.005	
	Min, Max	0.0045–15.13	0.06–4906.87	

A significant difference in RAF1 expression was observed between the patient and control groups. The relationship between the clinicopathological characteristics of AML patients and the expression levels of RAF1, miR-146b-3p, and Circ-RPL15 was evaluated. Ultimately, it was found that changes in RAF1 expression were significantly associated with gender (χ² p-value: 0.023, OR: 0.16, 95 % CI: 0.031–0.825). However, no significant associations were found between changes in miR-146b-3p or Circ-RPL15 expression and clinicopathological features.

Additionally, no significant correlations were found between the expression levels of RAF1, miR-146b-3p, and Circ-RPL15 with one another.

### 3.2 Evaluation of diagnostic value

**Fig. 1:**
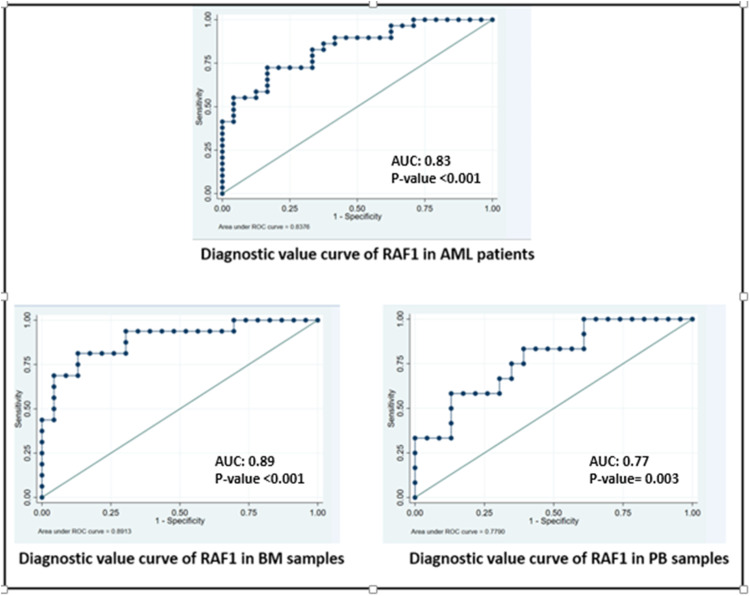
ROC curve in examining the diagnostic value of RAF1 in total and sample separation.

To assess the diagnostic value of RAF1, miR-146b-3p, and Circ-RPL15 expression as biomarkers in AML patients, ROC curves were plotted (Fig. 1) for different sample types (bone marrow and peripheral blood) as well as for the total sample set. Among the network components, only RAF1 demonstrated excellent diagnostic value in both total samples and in bone marrow and peripheral blood samples (Table 3).

**Table 3:** Diagnostic analysis for RAF1 in AML, also, in bone marrow and peripheral blood samples.

Diagnostic indicators	AUC (95 %CI)	P-value	Sen (95 %CI)	Spe (95 %CI)	DOR (95 %CI)	PLR (95 %CI)	NLR (95 %CI)	PPV % (95 %CI)	NPV (95 %CI)	Youden	Cut of point
RAF1 AML vs. controls	0.83 (0.73–0.94)	<0.001	0.72 (0.56–0.88)	0.83 (0.68–0.98)	13.16	4.34 (1.72–10.92)	0.33 (0.17–0.61)	0.84 (0.69–0.98)	0.71 (0.54–0.88)	1.55	1.50
In BM AML vs. controls	0.89 (0.78–0.99)	<0.001	0.81 (0.62–1.00)	0.87 (0.73–1.00)	29.61	6.22 (2.11–18.36)	0.21 (0.07–0.60)	0.81 (0.62–1.00)	0.87 (0.73–1.00)	1.68	1.50
In PB AML vs. controls	0.77 (0.61–0.90)	0.003	0.58 (0.30–0.86)	0.87 (0.73_1.00)	9.51	4.47 (1.40–14.24)	0.47 (0.24–0.95)	0.70 (0.41–0.98)	0.80 (0.64–0.95)	1.45	1.57

### 3.3 Survival analysis of RAF1

**Fig. 2:**
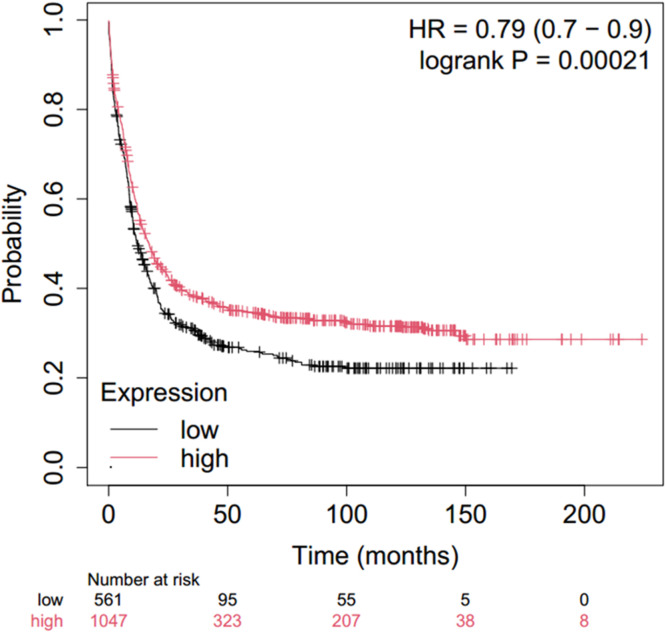
Kaplan-Meier for survival analysis in AML patients in correlation with RAF1.

The Kaplan-Meier Plot was meticulously constructed to evaluate the impact of RAF1 expression levels on the overall survival rates of patients diagnosed with AML. The graphical representations were generated utilizing expression data derived from a cohort of 1608 patients, sourced from the datasets GSE1159, GSE12417, GSE37642, GSE6891, and GSE8970. The findings indicated a statistically significant correlation between the expression levels of RAF1 and the overall survival of AML patients, yielding a log-rank value of 0.0002 and a hazard ratio of 0.79 (with a confidence interval ranging from 0.7 to 0.9) (Fig. 2).

## 4 Discussion

### 4.1 Biomarkers in disease diagnosis and monitoring

Biomarkers are measurable and reproducible indicators that can be used to assess and monitor an individual’s health or disease status [13,14].In recent years, extensive research has focused on microRNAs as biological biomarkers for diagnosing various cancers. These biomarkers are accessible and measurable by collecting a small amount of blood from the patient, a relatively inexpensive and minimally invasive method [[15], [16], [17], [18]]. Key characteristics of an ideal biomarker include ease of measurement, cost-effectiveness, and repeatability [19,20].Blood collection and microRNA analysis meet all these criteria, making them effective tools for health monitoring and disease diagnosis.

In 2018, Drobna et al. conducted a seminal study highlighting the role of microRNAs in T-cell acute lymphoblastic leukemia (T-ALL). This study emphasized that microRNAs can act as both tumor suppressors and oncogenes in T-ALL, depending on their target genes. It also highlighted the role of miR-146a/b in negatively regulating T-cell activation and influencing T-ALL progression through their effects on immune responses. The findings underscored the integral role of microRNAs in T-ALL biology and their potential as biomarkers for diagnosis, prognosis, and therapeutic targeting [21].

The relationship between miR-146b-3p and TNFAIP2 protein (Tumor Necrosis Factor Alpha-Induced Protein 2) in the context of AML was explored in another study. The key findings demonstrated that enhanced expression of TNFAIP2 or the knockdown of miR-146b-3p significantly induced differentiation in MOLM-13 cells (a human leukemia cell line). This study provided evidence that the miR-146b-3p/TNFAIP2 axis plays a fundamental role in regulating cellular differentiation in AML [22].

Similarly, another study showed that suppression of circ-RPL15 inhibits the RAS/RAF1/MEK/ERK pathway via miR-146b-3p This interaction indicates a connection between RAF1, miR-146b-3p, and Circ-RPL15 in chronic lymphocytic leukemia (CLL) [11].

### 4.2 Diagnostic and prognostic significance of RAF1, miR-146b-3p, and Circ-RPL15 in AML

This study investigated the expression levels of RAF1, miR-146b-3p, and Circ-RPL15 in AML patients compared to controls. Regardless of sample type (peripheral blood or bone marrow), RAF1 showed a 34.5-fold increased expression in the AML group compared to the control group, demonstrating high diagnostic value as a biomarker for AML. Assessing peripheral blood samples showed significant diagnostic results with excellent predictive power. Similarly, bone marrow samples highlighted the exceptional ability of RAF1 to distinguish AML patients from healthy controls. The high sensitivity and specificity of RAF1 expression in bone marrow samples further underscore its utility as a strong and reliable biomarker for AML diagnosis. Of course, it is important to note that this result was obtained from this study and generalization to all patients with AML requires further research and clinical trials.

Like our study, a related study assessed the diagnostic and prognostic significance of RAF1 alongside miR-106a in breast cancer. The findings confirmed that both markers are crucial for early detection and management of breast cancer, further emphasizing RAF1′s diagnostic potential [23]. Survival studies yielded promising results for the identification of RAF1 as a valuable prognostic biomarker in AML. It is important to note that, although the analysis of gene expression levels can provide valuable insights into the molecular basis of AML and may pave the way for the discovery of novel and effective diagnostic and prognostic biomarkers, it cannot serve as a substitute for current diagnostic and prognostic methods. Moreover, the validation of the findings from this study as definitive and conclusive evidence requires extensive and long-term clinical investigations.

**Key Diagnostic Metrics:**

• **Diagnostic Odds Ratio (DOR):** The DOR of RAF1 in bone marrow samples was 29.61, indicating a very high diagnostic power for differentiating AML samples from healthy controls. A higher DOR signifies better diagnostic performance.
• **Positive Likelihood Ratio (PLR):** PLR measures how much more likely a positive test result is in diseased individuals than in healthy ones. A high PLR indicates a strong diagnostic test, as seen in this study.
• **Negative Likelihood Ratio (NLR):** NLR assesses the probability of a negative test result in individuals without the disease. Lower NLR values enhance the reliability of a test in ruling out disease.
• **Positive Predictive Value (PPV):** PPV reflects the probability that a positive test result truly indicates the presence of disease.
• **Negative Predictive Value (NPV):** NPV indicates the likelihood that a negative test result genuinely confirms the absence of disease.

## 5 Conclusion

This study was the first to explore the expression, relationships, diagnostic and prognostic value of RAF1, miR-146b-3p, and Circ-RPL15 in AML. Although no strong correlations were observed among the components of this network, RAF1 emerged as a highly valuable biomarker for diagnosis and prognosis of AML in both peripheral blood and bone marrow samples. By identifying specific biomarkers, clinicians can design more targeted and effective treatments tailored to the clinical and genetic profiles of individual patients.

## CRediT authorship contribution statement

**Seyedeh Kosar Rahimpour:** Software, Formal analysis, Writing – original draft, Investigation, Project administration. **Yasin Mirazimi:** Methodology. **Seyedeh Zahra Shahrokhi:** Writing – review & editing. **Mohammad Sayyadi:** Writing – review & editing. **Maryam Kargar:** Writing – review & editing. **Mohammad Rafiee:** Validation, Conceptualization, Investigation, Supervision.

## Declaration of competing interest

The authors declare the following financial interests/personal relationships which may be considered as potential competing interests:

Mohammad Rafiee reports financial support was provided by Zanjan University of Medical Sciences. Mohammad Rafiee reports a relationship with Zanjan University of Medical Sciences that includes: board membership. Mohammad Rafiee has patent pending to A-12–1757–1 and A-12–1757–8. I have nothing to declare. If there are other authors, they declare that they have no known competing financial interests or personal relationships that could have appeared to influence the work reported in this paper.
